# Evaluation and validation of reference genes for RT-qPCR normalization in different sweet potato tissues

**DOI:** 10.1038/s41598-025-22650-7

**Published:** 2025-11-14

**Authors:** Melissa Barbosa Fonseca Moraes, Matheus Martins Daúde, Kellen Kauanne Pimenta de Oliveira, Rogério Cavalcante Gonçalves, Solange Aparecida Ságio, André Almeida Lima, Antonio Chalfun-Junior, Márcio Antônio da Silveira, Horllys Gomes Barreto

**Affiliations:** 1 Laboratory of Molecular Analysis (LAM), Life Sciences Department, Federal University of Tocantins, Palmas, TO Brazil; 2 Postgraduate Program in Biodiversity and Biotechnology, Rede Bionorte, Federal University of Tocantins, Palmas, TO Brazil; 3 Postgraduate Program in Digital Agroenergy, Federal University of Tocantins, Palmas, TO Brazil; 4 Plant Molecular Physiology Laboratory, Biology Department, Federal University of Lavras, Lavras, MG Brazil

**Keywords:** Biological techniques, Biotechnology, Molecular biology

## Abstract

Sweet potato (*Ipomoea batatas*) is one of the most important crops of the world, displaying a significant economic importance in several countries. However, few studies exploring biotechnological tools, such as RT-qPCR, used for gene expression analysis, have been conducted so far, slowing down crop breeding programs. Here, a detailed analysis of previous validated reference genes, essential for normalization of RT-qPCR studies, was conducted, and new candidate genes were evaluated, addressing existing gaps for the development of molecular studies via RT-qPCR in sweet potato. To this end, five sweet potato reference genes studies were evaluated, and the six best-classified genes (*IbCYC*, *IbARF*, *IbTUB*, *IbUBI*, *IbCOX* and *IbEF1α*) were selected for further analysis. Additionally, four commonly used reference genes (*IbPLD*, *IbACT*, *IbRPL* and *IbGAP*) were also included in the study. The ten reference genes were analyzed across four different tissues (fibrous root, tuberous root, stem and leaf) from sweet potato plants grown under normal conditions. *IbACT*, *IbARF* and *IbCYC* were the most stable genes, displaying the lowest variation in expression levels across the tissues studied, while *IbGAP*, *IbRPL* and *IbCOX* were classified as the least stable genes according to the RefFinder algorithm. The results obtained here highlight the relevance of this type of investigation to ensure the reliability of relative gene expressions analyzed in sweet potato. In addition, it directly contributes for a better understanding of the biological processes associated with the performance of this crop, aiding future research addressing transcriptome analyses in this species, which displays high agricultural and industrial potential.

**Supplementary Information:**

The online version contains supplementary material available at 10.1038/s41598-025-22650-7.

## Introduction

Sweet potato (*Ipomoea batatas* (L.) *Lam*.) is a dicotyledonous species, from the *Convolvulaceae* family, adapted to tropical and subtropical climates. It is among the leading plant species used for human nutrition worldwide^1^. Sweet potato tubers are an excellent source of nutrients, rich in vitamin A, *β*-carotene, anthocyanins, proteins and minerals^2,3^, and its leaves and shoots are also used as animal feed^4^. In addition to its nutritional value, sweet potato has applications in the development of pharmaceutical products, phytochemicals^5^, distillates products, and energy generation^6^.

Being cultivated in several countries and reaching a total global production of 93.5 million tonnes, China stands out as the world leader in sweet potato production, accounting for approximately 51.4 million tonnes. Other important producers include Malawi (8.0 million tonnes), Tanzania (4.5 million tonnes), and Nigeria (4.1 million tonnes)^7^, highlighting the economic importance of this crop worldwide.

Thus, due to its economic and nutritional importance, sweet potato genetic improvement is essential to maximize yield and promote the sustainable use of this crop. However, this process is challenging since sweet potato is a hexaploid species (2n = 6x = 90 chromosomes)^8,9^, which makes traditional breeding a complex process. In this context, biotechnological techniques have become indispensable to complement classical plant-breeding methods, enabling the development of genotypes with enhanced resistance and tolerance to biotic and abiotic stresses. Moreover, these techniques contribute to a better understanding of the physiological responses of plants to different environmental conditions through the study of genes, thus fostering significant advances in production systems, from quality to productivity.

Among the most commonly used gene expression analysis techniques, real-time PCR (RT-qPCR) stands out, mainly due to its high sensitivity, specificity, speed, and reproducibility^10–13^. However, this method requires the careful observation and optimization of some parameters in order to guarantee the quality of the results, such as RNA integrity and concentration, cDNA quality, number of repetitions, amplification efficiency, and the adequate choice of reference genes when dealing with relative expression projects, where these genes are used to normalize the data obtained^13–18^.

The best reference genes are those that exhibit the highest expression stability, that is, minimal variation across different tissues and/or experimental conditions^19,20^. Therefore, it is important to select and validate adequate reference genes, based on their expression stability in different experimental conditions, in order to guarantee the quality of the results, since the use of inappropriate reference genes affects the precision and reliability of the results^16–18,21,22^. Several studies have previously described the selection of reference genes associated with different abiotic and biotic stress conditions in sweet potato^20,22–24^. However, these studies were conducted independently, and focused on specific tissues or experimental conditions, not providing a comprehensive evaluation that integrates the most relevant genes across different sweet potato tissues under normal conditions. In the present study, we not only selected the top-performing reference genes identified in previous studies but also validated their expression stability under our experimental conditions, enabling the identification of the most suitable candidates.

Considering the economic and nutritional importance of sweet potato cultivation and the valuable information that can be obtained through transcriptional studies, this study aimed to evaluate the stability of sweet potato reference genes previously identified in earlier studies (*IbCYC*, *IbARF*, *IbTUB*, *IbUBI*, *IbCOX* and *IbEF1α*), as well as commonly used plant reference genes (*IbPLD*, *IbACT*, *IbRPL* and *IbGAP*) in this crop. The analyses were performed on fibrous roots, tuberous roots, stems, and leaves of plants grown under normal conditions, and the RefFinder algorithm^25,26^, which integrates GeNorm^27^, NormFinder^28^, BestKeeper^29^, and Delta-Ct^30^, was used to select the most suitable reference genes.

## Results

### Expression level of candidate reference genes

**Fig. 1:**
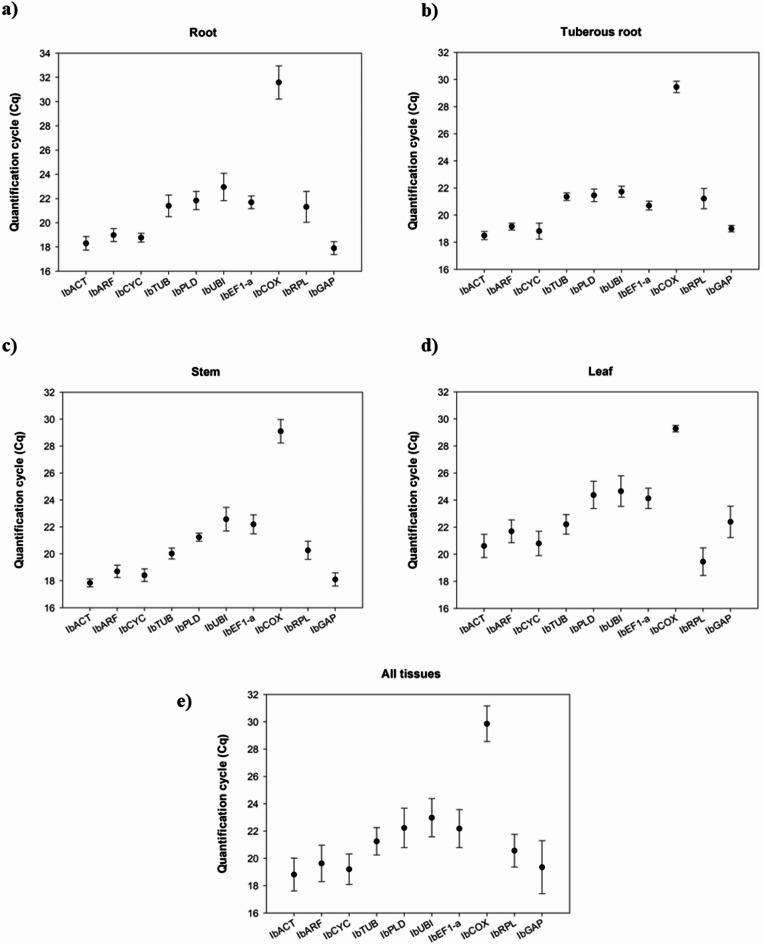
Expression levels of the candidate reference genes based on the Cq (Cycle of Quantification) data obtained from fibrous roots (**a**), tuberous roots (**b**), stem (**c**), leaves (**d**), and the combination of the four previous mentioned tissues (**e**) from sweet potato (*Ipomoea batatas*) plants grown under natural conditions. Vertical bars represent the standard deviation, and the black dots represent the mean Cq values.

The individual analysis of the mean Cq values for each tissue allowed the observation that in fibrous roots the most expressed genes were *IbGAP*, *IbACT* and *IbCYC*, with mean Cq values of 17.91, 18.31 and 18.77 (Fig. 1a). For tuberous roots, *IbACT*, *IbCYC* and *IbGAP* showed the highest expression levels, with mean Cq values of 18.49, 18.82 and 19.01, respectively (Fig. 1b). Similarly, the same group of genes was found to be most expressed in the stem at the following order: *IbACT*, *IbGAP* and *IbCYC*, with Cq values of 17.84, 18.10 and 18.41 (Fig. 1c). On the other hand, *IbRPL* showed the highest expression level in leaves (Cq = 19.45), being followed by the genes *IbACT* (Cq = 20.61) and *IbCYC* (20.80).

In relation to the least expressed genes, the individual tissue analysis showed that *IbCOX*, *IbUBI* and *IbPLD* displayed the lowest expression levels in fibrous roots and tuberous roots, with average Cq values of 31.58, 22.94 and 21.83 in fibrous roots and 29.45, 21.73 and 21.46 in tuberous roots, respectively (Fig. 1a). Similarly, the genes *IbCOX* and *IbUBI* showed the lowest expression levels in stems, with average Cq values of 29.10 and 22.57, respectively, followed by *IbEF1α*, with an average value of 22.10 (Fig. 1c). Finally, for leaves (Fig. 1d), the least expressed genes were *IbCOX*, *IbUBI* and *IbPLD*, with average Cq values of 29.28, 24.66 and 24.38, respectively.

When all tissues are analyzed together, it could be observed that the 10 candidate reference genes showed a significant variation in their expression, displaying mean Cq values from 19 to 30 (Fig. 1e). The mean Cq values analysis indicated that *IbACT*, *IbCYC* and *IbGAP* showed the highest expression levels for the analyzed tissues, with mean Cq values of 18.81, 19.20 and 19.35 respectively. On the other hand, *IbCOX*, *IbUBI* and *IbPLD* showed the lowest expression levels, with mean Cq values of 29.85, 22.98 and 22.23 respectively.

### Expression stability of the candidate reference genes

**Fig. 2:**
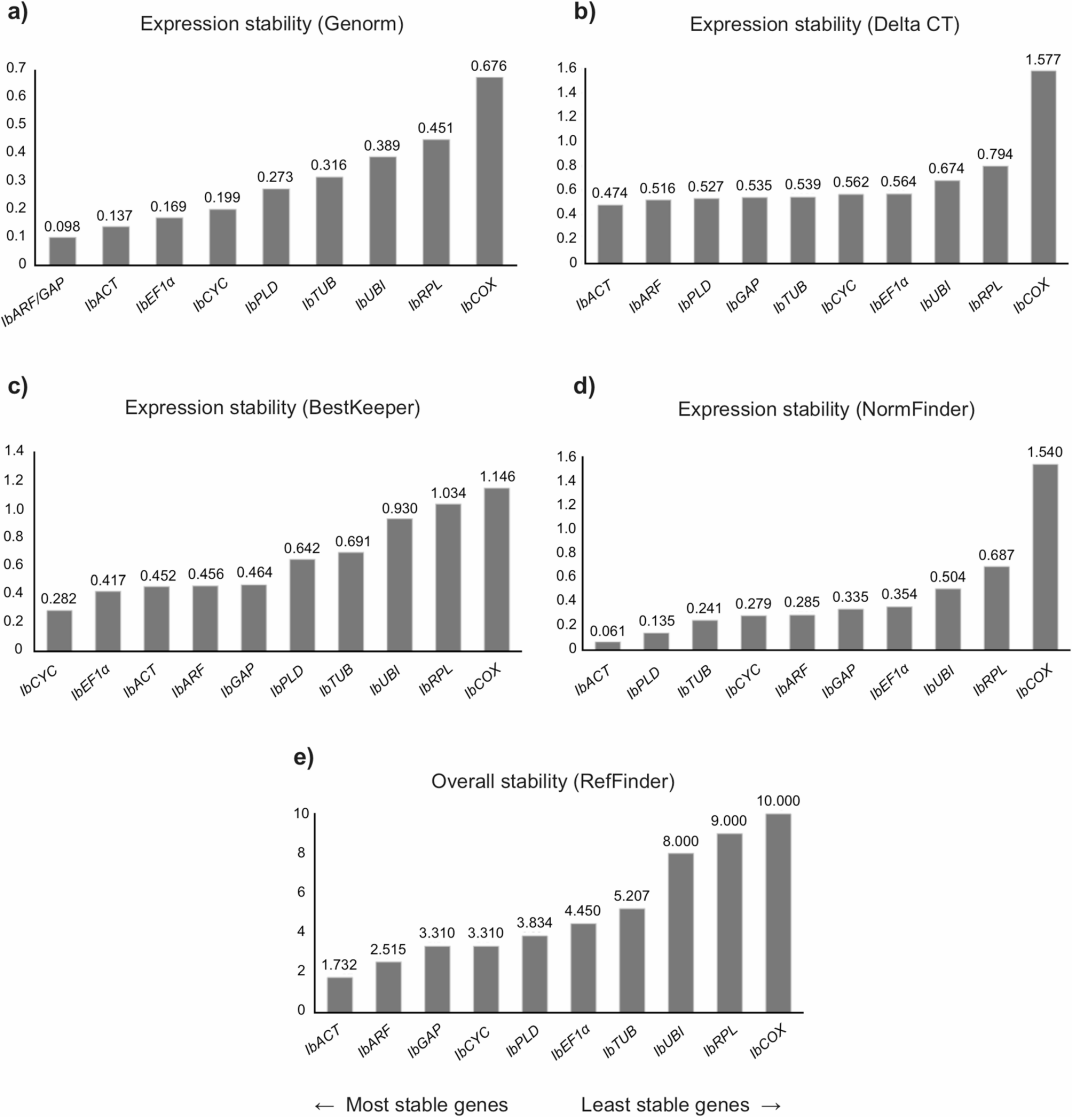
Ranking of the candidate reference genes generated according to their stability values calculated by the geNorm (**a**), Delta-Ct (**b**), BestKeeper (**c**), NormFinder (**d**), and RefFinder (**e**) algorithms, using the Cq (Cycle of Quantification) values obtained from fibrous roots of sweet potato plants grown under normal conditions.

**Fig. 3:**
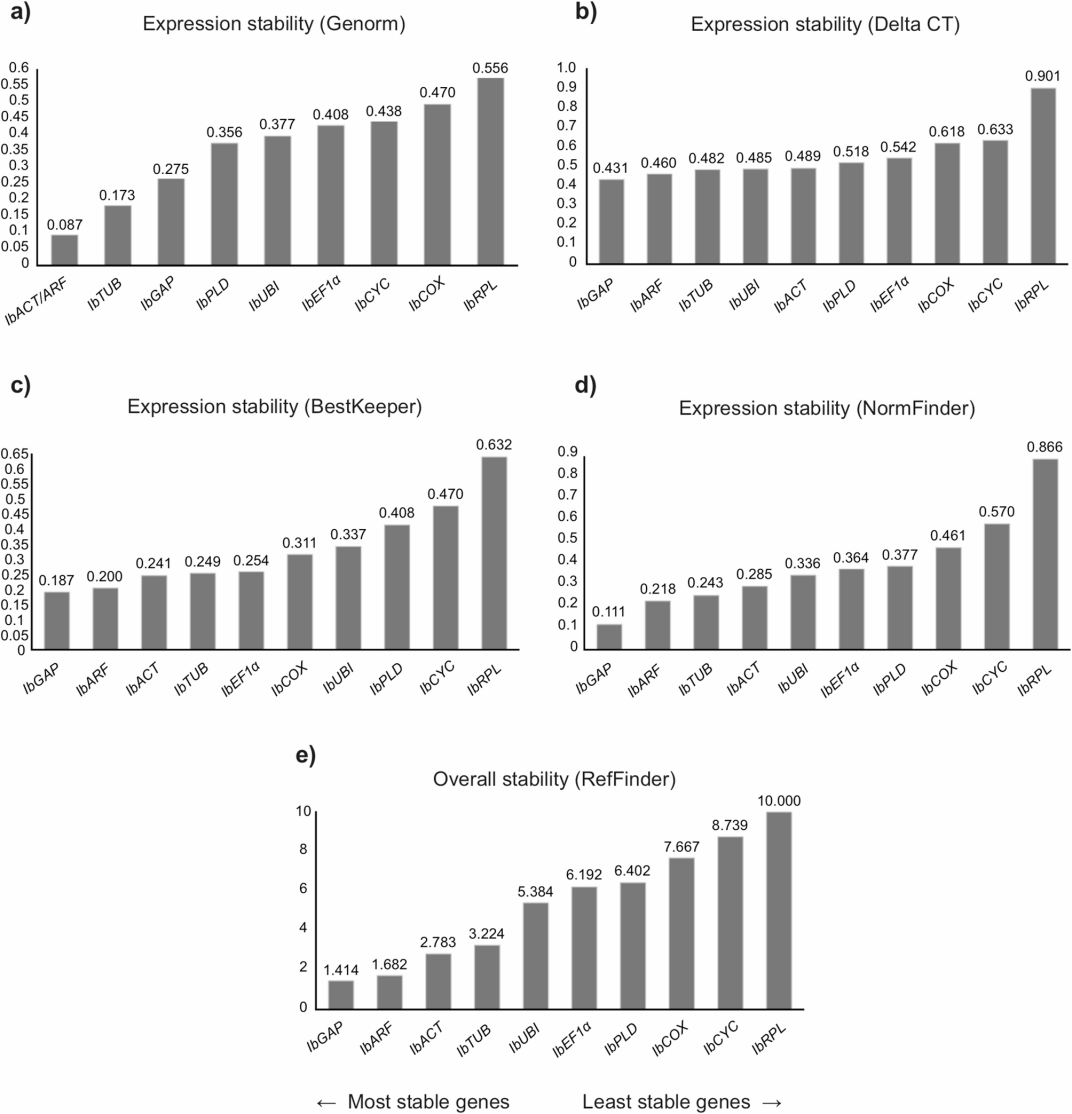
Ranking of the candidate reference genes generated according to their stability values calculated by the geNorm (**a**), Delta-Ct (**b**), BestKeeper (**c**), NormFinder (**d**), and RefFinder (**e**) algorithms, using the Cq (Cycle of Quantification) values obtained from tuberous roots of sweet potato plants grown under normal conditions.

**Fig. 4:**
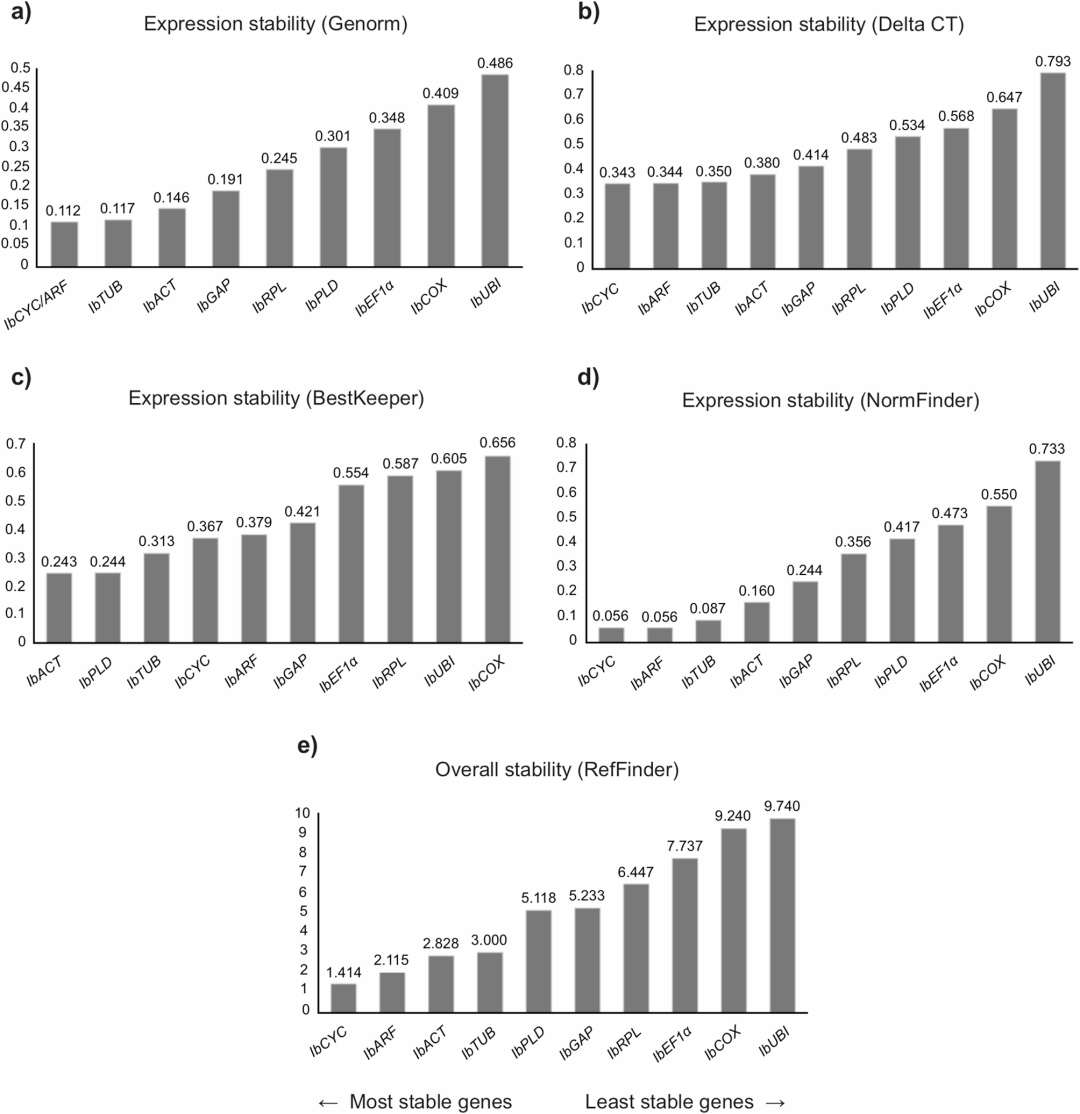
Ranking of the candidate reference genes generated according to their stability values calculated by the geNorm (**a**), Delta-Ct (**b**), BestKeeper (**c**), NormFinder (**d**), and RefFinder (**e**) algorithms, using the Cq (Cycle of Quantification) values obtained from the stem of sweet potato plants grown under normal conditions.

**Fig. 5:**
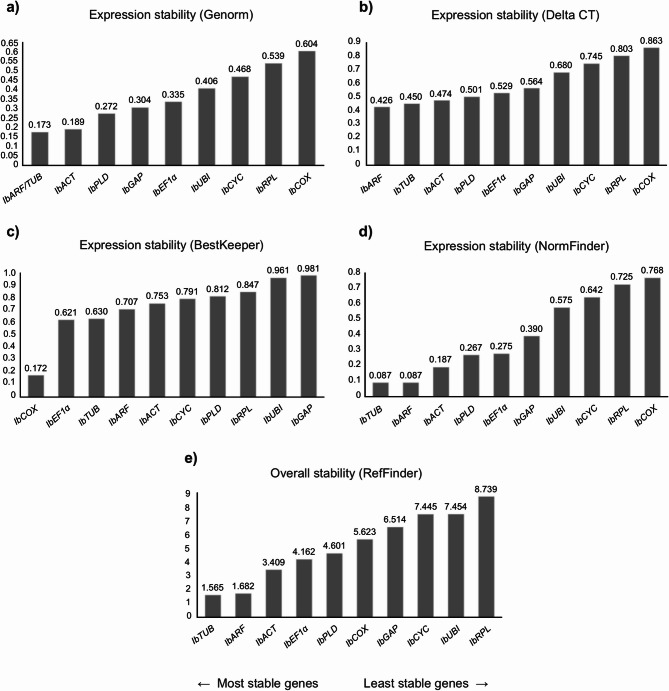
Ranking of the candidate reference genes generated according to their stability values calculated by the geNorm (**a**), Delta-Ct (**b**), BestKeeper (**c**), NormFinder (**d**), and RefFinder (**e**) algorithms, using the Cq (Cycle of Quantification) values obtained from leaves of sweet potato plants grown under normal conditions.

**Fig. 6:**
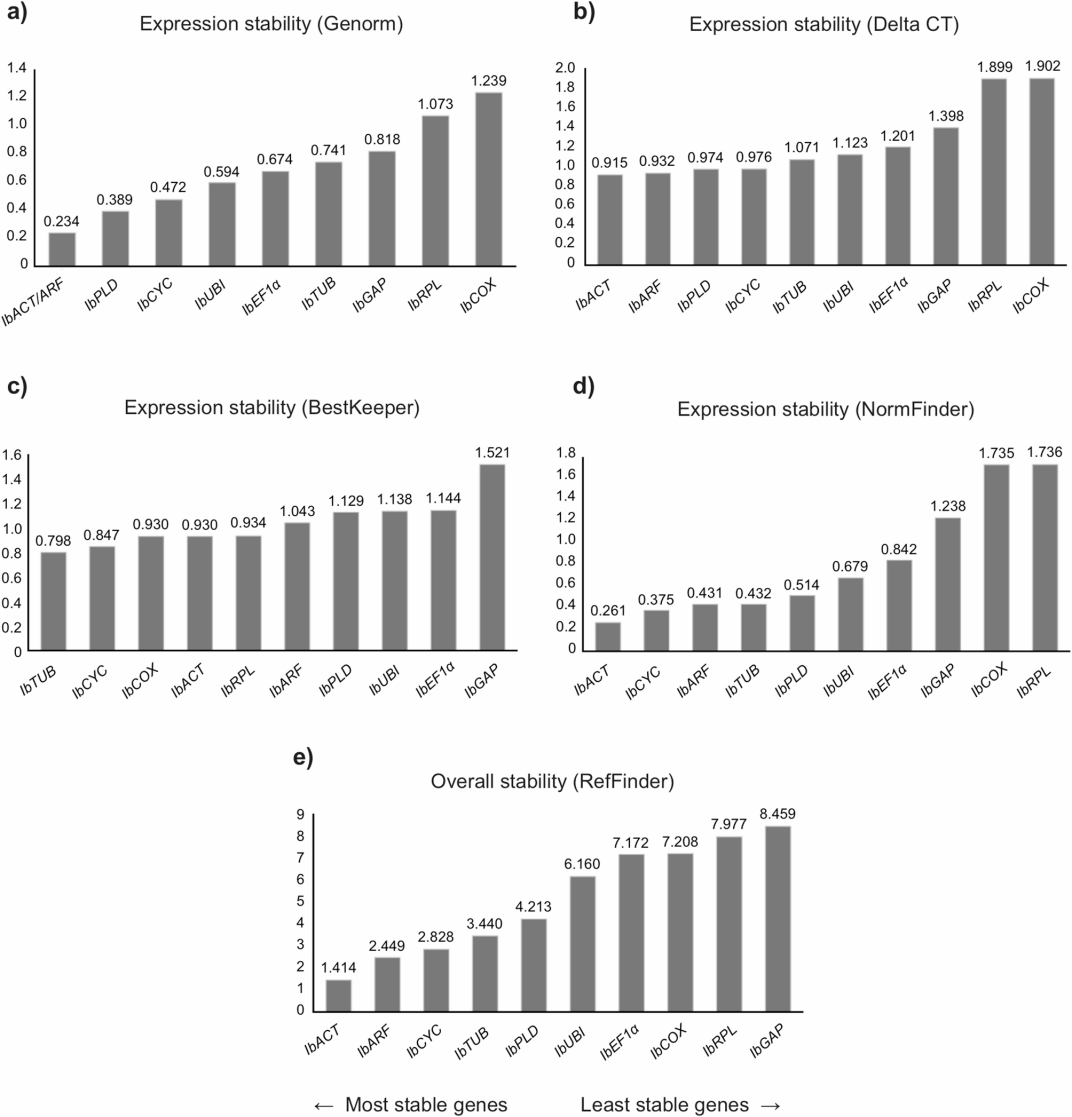
Ranking of the candidate reference genes generated according to their stability values calculated by the geNorm (**a**), Delta-Ct (**b**), BestKeeper (**c**), NormFinder (**d**), and RefFinder (**e**) algorithms, using the Cq (Cycle of Quantification) values of the four sweet potato tissues (fibrous roots, tuberous roots, stem, and leaves) combined.

The analysis of the expression stability of the candidate reference genes was performed using the geNorm, NormFinder, BestKeeper, and Delta-Ct algorithms, and by RefFinder, which integrates the results of the four previously mentioned algorithms. This analysis was conducted on all analyzed tissues, considering every possible combination. The results are presented in Figs. 2, 3, 4, 5 and 6, in the supplementary material.

### Fibrous roots

The expression analysis of the candidate reference genes in fibrous roots showed that *IbACT* was among the three most stable genes in three of five tested algorithms: NormFinder, Delta-Ct and RefFinder (Fig. 2). Furthermore, the *IbARF* and *IbGAP* were identified as more stable by geNorm, while for NormFinder it was the *IbPLD* and *IbTUB* genes. For the BestKeeper algorithm, *IbCYC* and *IbEF1α* were the most stable reference genes, while *IbARF* and *IbPLD* were the most stable according to the Delta-Ct algorithm. The overall classification by RefFinder reinforced the stability of *IbACT*, *IbARF* and *IbGAP* as the main root reference genes. On the other hand, *IbCOX*, *IbRPL* and *IbUBI* were considered the least stable genes.

### Tuberous roots

The evaluation of the candidate reference genes in tuberous roots showed that for the algorithms NormFinder and Delta-Ct, *IbGAP*, *IbARF* and *IbTUB* were the most stable genes, while for geNorm and BestKeeper *IbARF* and *IbACT* were classified the genes with higher stability, being followed by *IbTUB* and *IbGAP* for geNorm and BestKeeper, respectively (Fig. 3). In contrast, *IbRPL* and *IbCYC* were identified as the least stable genes in the four algorithms analyzed, with *IbCOX* and *IbPLD* completing the ranking of the three genes with the lower stability values for geNorm, NormFinder, and Delta-Ct, and BestKeeper, respectively. The general ranking generated by RefFinder showed that *IbGAP*, *IbARF* and *IbACT* were classified as the most stable genes, while *IbRPL*, *IbCYC* and *IbCOX* were shown to be the most variable genes and, therefore, the least recommended reference genes.

### Stems

In relation to sweet potato stem, *IbCYC*, *IbARF* and *IbTUB* were identified as the most stable genes according to geNorm, NormFinder and Delta-Ct algorithms (Fig. 4), while for the BestKeeper algorithm, *IbACT*, *IbPLD* and *IbTUB* were classified as the most stable genes. On the other hand, *IbUBI* and *IbCOX* were consistently ranked as the least stable genes in all four algorithms used, along with *IbEF1α* for geNorm, NormFinder and Delta-Ct, and *IbRPL* for BestKeeper. In the general evaluation by RefFinder, the best genes were *IbCYC*, *IbARF* and *IbACT*, while for the least stable genes, similar to geNorm, NormFinder and Delta-Ct algorithms, *IbUBI*, *IbCOX*, *IbEF1α* were classified as the most variables genes.

### Leaves

In sweet potato leaves, the algorithms geNorm, NormFinder and Delta-Ct classified *IbTUB*, *IbARF* and *IbACT* as the most stable genes (Fig. 5). On the other hand, BestKeeper identified *IbCOX*, *IbEF1α* and *IbTUB* as the best reference genes. In relation to the most variable genes, for geNorm, NormFinder and Delta-Ct, *IbCOX* and *IbCYC* were defined as the least stable genes, and *IbRPL* was the third least stable gene in all four algorithms analyzed. Differently from the other algorithms, BestKeeper identified *IbGAP* and *IbUBI* as the worst reference genes. The general analysis performed by the RefFinder algorithm classified *IbTUB*, *IbARF* and *IbACT*, and *IbRPL*, *IbUBI* e *IbCYC* as the most and least stable genes, respectively.

### Overall tissue analysis

Cq values from the four sweet potato tissues evaluated were combined to analyze the overall expression stability of the candidate reference genes (Fig. 6). *IbACT* and *IbARF* were classified among the three most stable genes by geNorm, NormFinder and Delta-CT algorithms. *IbCYC* was considered one of the least variable genes by NormFinder and BestKeeper, while *IbPLD* was among the best three reference genes classified by geNorm and Delta-CT. Unlike the other algorithms, Bestkeeper ranked *IbTUB* and *IbCOX* as the most stable genes. Regarding the most variable genes, *IbCOX*, *IbRPL* and *IbGAP* were classified as the least stable genes by geNorm, NormFinder and Delta-Ct. For BestKeeper, the worst reference genes were *IbGAP*, *IbEF1α* and *IbUBI*. RefFinder classified *IbACT*, *IbARF* and *IbCYC* as the genes with lower expression stability, while *IbGAP*, *IbRPL* and *IbCOX* were classified as the least stable genes.

### Reference gene validation

In order to assess the impact of reference gene selection, the relative expression of the target gene *IbAGPASE* was analyzed. This gene encodes for the ADP-glucose pyrophosphorylase (AGPase; EC: 2.7.7.27) enzyme, which is involved in starch biosynthesis, displaying a crucial role for tuberous root development^31^. The expression data were normalized using the candidate reference genes previously evaluated for their stability in the different sweet potato tissues analyzed in this study.

**Fig. 7:**
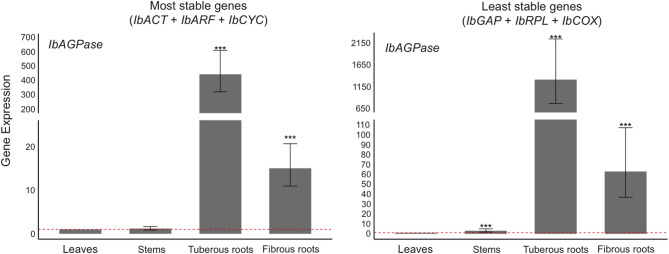
*IbAGPASE* expression profile when normalization was conducted by using the most stable (*IbACT*, *IbARF*, and *IbCYC*) or least (*IbGAP*, *IbRPL*, and *IbCOX*) stable reference genes, as determined by the RefFinder algorithm, in leaves, stems, tuberous roots, and fibrous roots of sweet potato plants grown under normal conditions. Bars represent fold change (FC) in gene expression relative to the reference sample (leaves). Expression levels are based on three biological replicates, and error bars represent the 95% confidence interval calculated using the LMM methodology. Asterisks denote significance levels: *** *P* < 0.001, ** *P* < 0.01, and * *P* < 0.05 (n = 3). Contrasts were considered statistically significant when the confidence interval did not cross the cutoff of 1 (dashed red line).

The results revealed that the *IbAGPASE* expression profile substantially varied depending on the reference genes selected for normalization (Fig. 7). When *IbAGPASE* expression data were normalized using the most stable reference genes, expression in tuberous roots was 29-fold higher than in fibrous roots, a statistically significant difference. When normalization was performed using the least stable reference genes, *IbAGPASE* expression in tuberous roots was still higher, however the fold-change difference dropped to 21, also statistically significant.

Analysis of *IbAGPASE* expression in other two sweet potato tissues, stem and leaves, showed no statistically significant difference when the most stable reference genes were used for normalization. However, when the least stable reference genes were employed, *IbAGPASE* expression was statistically higher in stems compared to leaves (Fig. 7).

## Discussion

The use of reference genes is widely recognized as the most common and recommended method for normalizing relative gene expression data^13,26,32^, which enables the study of biological processes such as flowering^33,34^ and somatic embryogenesis^35,36^. Normalization is a crucial step to correct variations that may originate from different stages of the analysis, such as collection of biological material, RNA extraction and cDNA synthesis^29,32^. Relative gene expression calculations, as described by Livak and Schmittgen^37^ and Pfaffl^38^, necessarily include data from reference genes in their equations. Therefore, the appropriate selection of reference genes is essential for accurate, reproducible and reliable performance of gene expression studies, being widely studied in different organisms and experimental conditions^18,39–41^.

The careful selection of these genes, as carried out in this study, represents a crucial preliminary step in gene expression studies, as inappropriate reference genes can result in erroneous inferences of target genes expression^18,42^. Although previous studies have been conducted on reference genes for sweet potato^20,23,24^, the present study stands out by incorporating the most suitable genes previously reported in the literature into a detailed analysis, along with other reference genes commonly used for gene expression normalization in plants.

As shown in Table 1, our results not only confirm the stability of certain sweet potato reference genes, such as the *IbARF* gene, but also provide new insights into the selection of reference genes for studying the four tissues analyzed in plants grown under normal conditions, including the *IbACT* and *IbCYC* genes, validated in this study. By addressing this gap, our work establishes a more robust foundation for gene expression studies in sweet potato, ensuring greater reliability and reproducibility in future gene expression analyses.

**Table 1:** Overview of the most suitable reference genes for gene expression studies in sweet potato (*Ipomoea batatas*) using RT-qPCR, including cultivar, tissue types, experimental conditions, genes analyzed, most and least stable genes, algorithms used, and references.

Cultivar	Tissue	Experimental condition	Genes	Most stable genes	Least stable genes	Algorithm	References
Yulmi, Sinzami, Sinhwangmi e Whitestar	Leaves, petioles, stems, fibrous roots, pencil roots and storage roots	Cold stress, oxidative stress, salt stress and drought stress	*ACT*, *RPL*, *GAP*, *CYC*, *TUB*, *ARF*, *H2B*, *UBI*, *COX,* and *PLD*	*ARF, UBI and COX*	*RPL*, *H2B and* *ACT*	geNorm and NormFinder	Park et al.,^23^
–	Leaves, fibrous roots, storage roots, ovary and petals	Cold stress, heat stress and control (Leaf)	*ACT*, *ARF*, *COX*, *CYC*, *GAPDH*, *H2B1*, *PLD*, *RPL2*, *α-tubulin*, *UBI*, *β-tubulin*, *G14*, *elF*, *HIS*, *EF1-a*, and *UBQ*	*elF*	*UBI*	Delta-Ct, geNorm, NormFinder, BestKeeper	Yu et al.,^20^
XS-18 (Xushu18) and XZS-3 (Xuzishu3)	Leaves petioles, stems and roots	Drought stress, salt stress and control	*U6, 5S, miR159, miR164, miR168, miR172, miR482, miRn3, miRn29,* and *miRn60*	*miRn60 and miR482*	*U6,and* *5S*	geNorm, NormFinder and BestKeeper	Liu et al.,^63^
Fucaishu18, Fucaishu23 and Ornamental yellow (OY)	Leaves, stems and roots	Salt stress, osmotic stress, cold stress, heavy-metal stress, hormone stress (GA3) and different tissues (control)	*18srRNA*, *ACT*, *EF1α*, *eIF4α*, *GADPH*, *TIP41*, *TUA*, and *TUB*	*TUA* and *EF1α*	*eIF4α* and *GADPH*	Delta-Ct geNorm, NormFinder, BestKeeper, and RefFinder	Guoliang et al.,^24^
Jishu25	Leaves and roots	Virus-infected and nonvirus-infected samples	*ARF, GAP, PLD, UBI, ACT, 18S, ATUB, and CYP*	*PLD and GAP (infected)*	*–*	geNorm, NormFinder and Bestkeeper	Li et al.,^22^

### Reference genes expression level

The appropriate selection of reference genes is essential to ensure the robustness and reliability of gene expression analyses. Genes that are stable and moderately expressed (Cq values between 15 and 30) are preferred for normalization, guarantying that they can accurately reflect variations in RNA quantity and quality in a variety of biological samples^43^. Within this context, the results obtained in this study showed that the maximum and minimum Cq values in all tissues were within the recommended range (Fig. 1). These values are similar to the ones found in other sweet potato studies for the same tissues^20,22–24^. Among the 10 reference genes evaluated in this study, *IbACT* showed the lowest Cq value (18.81), while *IbCOX* displayed the highest Cq value (29.85).

### Reference gene expression stability

The results of the gene expression stability of the candidate reference genes revealed that, considering all tissues, *IbACT*, *IbARF* and *IbCYC* were the most stable reference genes, while *IbGAP*, *IbRPL* e *IbCOX* were classified as the least stable genes according to RefFinder (Fig. 6e). However, when all tissues are separately analyzed in each algorithm, the most stable genes were not the same (Fig. 6a–d).

It is important to highlight that *IbACT*, although not classified among the best reference genes in other studies conducted in sweet potato, here it was classified as the most stable gene when the four sweet potato tissues are considered in the analysis (Fig. 6e). However, in other plant species such as kiwi^44^, peach^45^ and banana^46^, *ACT* has been reported as one of the best reference genes under normal conditions, as well as in wheat seedlings under water stress^47^ and lychee fruits at different ripening stages^48^.

*IbARF*, classified as the second best reference gene in this study (Fig. 6e), has already been indicated as one of the best reference genes in previous studies in sweet potato under various conditions, such as cold stress, oxidative stress, saline stress,^23^, and also under biotic stress^22^.

These variations in reference gene expression stability may be related to differences in the algorithms used, which employ different stability calculation models, as well as the quality of the mRNA and types of treatments applied. It is important to highlight that in this study, the evaluated tissues were obtained from plants grown under normal conditions, without any type of treatment or stress, and the best genes for each single tissue, and for all tissues together, are recommended. These results emphasize the importance of context-specific selection of reference genes, as stability may significantly vary depending on the tissue type and environmental conditions being studied.

### Reference gene validation

The selection of adequate reference genes plays a crucial role in gene expression results, highlighting the importance of validating these genes. Furthermore, it is essential to select genes with a well-established expression pattern at this stage. In this context, the *IbAGPASE* gene was selected to validate the reference genes analyzed in this study. This gene encodes for the AGPase enzyme, whose activity is directly correlated to the dry matter of tuberous roots, resulting in an increase in starch accumulation and greater efficiency of the tuberization process^49^. Furthermore, recent studies indicate that this enzyme influences the regulation of carbohydrates in sweet potatoes^50^. AGPase catalyzes the first step of the starch metabolic pathway, converting ADP-glucose into glucose-1-phosphate, which is an essential precursor for starch synthesis^51,52^. Therefore, when comparing tissues such as leaves, stems and roots, it is expected to observe greater expression levels of this gene in tuberous root tissues.

The AGPase enzyme has already been identified in several plant species, including *Solanum tuberosum* (potato)^53^, *Zea mays* (corn)^54^, *Triticale sp.* (wheat)^55^, *Oryza sativa* (rice)^56^, *Manihot esculenta* (cassava)^57^ and *Ipomoea potatoes* (sweet potato)^31^, and *AGPASE* expression has been shown to be highly regulated during plant development and in response to various environmental stimuli, such as light, temperature and nutrient availability^58,59^. This fine-tuned regulation allows plants to control starch synthesis and storage according to their metabolism and environmental conditions^60^. Here, differences in the *IbAGPASE* expression pattern could be observed depending on the reference genes used during the normalization of the RT-qPCR data.

When normalization was carried out with the best (*IbACT*, *IbARF* and *IbCYC*) reference genes identified in this study (Fig. 3), the fold-change difference of *IbAGPASE* expression between tuberous roots and roots was higher (eight times) them the difference observed when the least (*IbGAP*, *IbRPL* and *IbCOX*) stable reference genes were used to normalize the results. These data corroborate with previous studies that used different tissues of sweet potato, at different developmental stages, and higher expression levels of this gene were also observed in tuberous roots tissue^50,61^. Similarly, in leaves and stems, although no differences in *IbAGPASE* expression were observed when the most stable reference genes were used to normalization, *IbAGPASE* expressed was higher in stems compared to leaves when the least stable reference genes were used for data normalization. On the other hand, Seo et al.^50^ analyzed the *IbAGPASE* expression in different sweet potato tissues and observed a higher expression level in the leaves, compared to the stems. These results confirm the influence of the reference genes used in the result of the RT-qPCR gene expression analysis, highlighting the importance of their adequate selection for the correct interpretation of the results.

## Conclusion

Based on results of the reference gene selection carried out here for sweet potato plants grown under normal conditions, we concluded that the most stable genes for RT-qPCR studies were *IbACT*, *IbARF* and *IbCYC*. Furthermore, choosing reference genes with lower stability levels may cause changes in the expression pattern of target genes, as observed for *IbAGPASE*. These results emphasize the importance of this type of research to increase the reliability of relative gene expression analyzes and can certainly aid in sweet potato transcriptome studies. Furthermore, this study establishes a solid basis for future gene expression research via RT-qPCR in this species, which has significant economic and social importance.

## Materials and methods

### Experiment design

#### Plant material

The experiment was conducted under field conditions at the Experimental Research Station of the Federal University of Tocantins (UFT), located in Palmas, Tocantins, Brazil. In this study, ‘normal conditions’ were defined as follows: a mean daily temperature of approximately 26 °C (with a daily range of ~ 22–35 °C), average relative humidity during the rainy season around 80%, a natural photoperiod consistent with equatorial light cycles (~ 12 h per day), and typical regional soil derived from the Cerrado biome (classified as Red-Yellow Latosol). Irrigation was applied only as a supplementary measure during the rainy season when rainfall was insufficient. Sweet potato plants of the ‘Duda’ cultivar, developed by the UFT’s breeding program, were used. Prior to planting, soil analysis was performed, and fertilization was applied following agronomic recomendations for sweet potato cultivation.

After 150 days from planting, samples of fibrous roots, tuberous roots, stems, and leaves were collected for gene expression analysis. Root samples were washed with running water to remove soil and other debris that could interfere in RNA extraction. All collected plant tissues were immediately frozen in liquid nitrogen and subsequently stored at − 80 °C until RNA extraction. For each tissue type, three biological replicates were used, with each replicate consisting of pooled material from four individual plants.

#### RNA extraction and cDNA synthesis

RNA extraction was performed using the CTAB (cetyltrimethylammonium bromide) method, according to Gonçalves et al.^62^. After extraction, the RNA quantity and purity (A_260_/A_280_ and A_260_/A_230_ ratios) were determined through a spectrophotometer (Nanodrop® One Spectrophotometer), while RNA integrity was verified using the agarose gel (1.0%). RNA samples (5 μg) were treated with DNase I, using the Turbo DNA-free kit (Ambion) and following its instructions, to eliminate residual DNA contamination. Subsequently, RNA was evaluated for its quantity and purity (A_260_/A_280_ and A_260_/A_230_ ratios) through spectrophotometry (Nanodrop® One Spectrophotometer) and its integrity was analyzed through agarose gels (0.8%). cDNA was synthesized from 1.0 μg of RNA using the High-Capacity cDNA Reverse Transcription kit (Applied Biosystems) and following the manufacturer’s protocol. cDNA samples were then stored at − 20 °C.

#### Reference gene identification and selection

The reference genes analyzed in this study were chosen from a literature search for sweet potato reference gene articles on the Web of Science database (www.webofknowledge.com), using the following keywords: housekeeping gene, endogenous gene, reference gene, sweet potato, and *Ipomoea batatas*. The Boolean interpolator “and” was used. From this search, the selection of the reference genes followed the recommendation criteria of the article in which they were analyzed, that is, the genes that had the best results (greater expression stability in the sample set used) were prioritized. It is important to mention that the study conducted by Lui et at ^63^. was not considered in the selection of references genes in this study, since it comprised only microRNAs, with these genes acting as reference genes only for small RNAs (sRNAs)^64^. Thus, six different genes, indicated as the best reference genes on their studies, were selected: *IbCYC* (cyclophilin), *IbARF* (diphosphate-ribosylation factor), *IbTUB* (tubulin), *IbUBI* (ubiquitin), *IbCOX* (cytochrome oxidase subunit Vc) e *IbEF1α* (elongation factor-1α alpha). In addition, four commonly used reference genes in plants were also included in the analysis: *IbPLD* (phospholipase D1 alpha), *IbACT* (actin), *IbRPL* (ribosomal Protein), *IbGAP* (glyceraldehyde-3-phosphate dehydrogenase).

#### In silico analysis—gene sequence identification

The reference and target gene sequences were obtained using the BLAST tool (Basic Local Alignment Search)^65^ through the comparison of their nucleotide sequences from sweet potato, obtained from the GenBank (http://www.ncbi.nlm.nih.gov/), with the Sweetpotato Genomics Resource a database (http://sweetpotato.plantbiology.msu.edu/) generated and made available by the University of Michigan/USA.

#### Primer design

RT-qPCR primers were designed by using the reference and target gene sequences obtained from the Sweetpotato Genomics Resource database and the OligoPerfect program (apps.thermofisher.com/apps/oligoperfect/), except for *IbTUB* and *IbACT* genes, which had their forward primer sequences obtained from the study conducted by Park et al.^23^, and for *IbCOX*, whose primer sequences were obtained from the study performed by Yu et al.^20^. Quality assessment of the designed primers was evaluated by the OligoAnalyzer tool (http://www.idtdna.com/calc/analyze) (Table 2).

**Table 2:** Primer information used for RT-qPCR, including gene name, accession number, primer sequences, melting temperature (Tm), amplicon size, correlation coefficient (R^2^), and amplification efficiency (E%) for the candidate reference genes and the analyzed target gene.

Gene name	Accession number	Primer sequence (5′-3′)	Tm (°C)	Amplicon (bp)	R^2^	E (%)
*IbTUB*	BM878762.1	Fw: TCCAAACCAACCTTGTACCC	62,1	149	0,996	87,0
		Rv: TTTTGCCATCATGCTTGAGG	61,4			
*IbACT*	EU250003.1	Fw: GTTATGGTTGGGATGGGACA	62,4	150	0,999	94,8
		Rv: GTTGTAGAAAGTGTGATGCCAG	61,4			
*IbARF*	JX177359.1	Fw: TGTTGGTGGTCAGGACAAGA	63,3	150	1,000	88,1
		Rv: CTCAATTCATCCTCATTCAGCAT	61,2			
*IbCYC*	EF192427.1	Fw: AACTTCATGTGCCAGGGCGG	67,2	156	0,999	94,4
		Rv: TGAAAGCCGTTGGTGTTGGGG	67,4			
*IbGAP*	JX177362.1	Fw: CGCTCACTTGAAGGCTGGT	64,4	151	0,999	91,7
		Rv: AGGAGCAAGGCAGTTGGTAG	63,9			
*IbPLD*	JX177360.1	Fw: CATTCCAGCATCCCGAAAGC	63,5	160	0,999	91,4
		Rv: AGCTCTGTTACGTCGCCATC	63,5			
*IbRPL*	AY596742.1	Fw: CCTTTGACCGAAATGCCCTT	63,1	159	0,998	88,0
		Rv: CAAACGGACCTCCCCAGAA	63,1			
*IbUBI*	JX177358.1	Fw: TCCACTCTCCACCTCGTCC	64,5	157	1,000	87,4
		Rv: GCCTCTGCACCTTTCCAGAC	64,5			
*IbEF1α*	HX977465.1	Fw: CTCCAAGGATGACCCAGC	60,3	109	1,000	91,7
		Rv: GGCAGTCGAGAACAGGAG	61,6			
*IbCOX*	S73602.1	Fw: CTCCCAGTGGCGGTGTTATG	63,0	111	0,999	86,8
		Rv: GGATGTTCTTGAGCCGGTCG	61,7			
*IbAGPaseASE*	AB271011.2	Fw: CCTCGCTTCTGGCAGATG	62,1	141	0,992	100,0
		Rv: GGCGGTCTGAGTCTTGAA	61,1			

#### RT-qPCR analysis

RT-qPCR analysis were carried out on an ABI PRISM 7500 Real-Time PCR thermocycler (Applied Biosystems), using the PowerUp™ SYBR™ Green Master Mix (Applied Biosystems) and the cDNA obtained in this study. Reactions were performed in 10 μL final volume: 1.0 μL of cDNA (diluted 1:5), 0.2 μL of each primer at 10 μM, and 5.0 μL of PowerUp™ SYBR™ Green Master Mix (Applied Biosystems), and 3.6 μL of RNase-DNase-free water. Three biological replicates were used, and reactions were run in triplicates as technical repetitions. Amplification reactions were carried out with the following conditions: 2 min at 50 °C, 5 min at 95 °C, followed by 40 cycles of 15 s at 95 °C and 1 min at 60 °C. In order to confirm the specificity of the primers, melting curves were generated after 40 amplification cycles for each primer pair by raising the temperature from 60 to 95 °C, with 1 °C increase in temperature every 5 s (Supplementary S2). Expression levels of candidate reference genes were established from the quantification cycle (Cq) values, with a fluorescence threshold set at 0.1. Expression data were normalized using more than one reference gene, in accordance with Bustin et al.^13^. For the *IbAGPASE* expression analysis, relative fold differences were calculated based on the ΔΔCT method^38^ and relative to a calibrator sample (leaves), which was selected because it showed the lowest expression level of *IbAGPASE* among the analyzed tissues. To evaluate the impact of reference gene stability on target quantification, normalization was performed by using the three most (*IbACT*, *IbARF*, and *IbCYC*) and least (*IbGAP*, *IbRPL*, and *IbCOX*) stable genes identified by RefFinder.

#### Expression stability analysis and reference gene validation

The algorithms geNorm^27^, NormFinder^28^, BestKeeper^29^ and Delta-Ct^30^ were used to calculate the reference gene stability values. Each algorithm generates a ranking based on its own stability metric. Subsequently, the results from these four algorithms were analyzed using the RefFinder tool (www.ciidirsinaloa.com.mx/RefFinder-master/), which integrates the rankings by assigning a weight to each gene according to its stability in each algorithms, and then calculating the geometric mean of these weights to provide an overall ranking of all candidate reference genes. This approach allows for a comprehensive assessment of gene stability, combining the strengths of the individual algorithms^25^. The following sample sets were used by the RefFinder tool to evaluate the stability of the 10 reference genes (*IbCYC*, *IbPLD*, *IbACT*, *IbARF*, *IbRPL*, *IbGAP*, *IbTUB*, *IbUBI*, *IbCOX* and *IbEF1α*) analyzed in this study: fibrous roots, tuberous roots, stem, leaves, and all tissues; as well as all possible combinations of these four studied tissues. These data can be accessed in the supplementary material of this study and, after publication, in the RGeasy database^66^ (http://rgeasy.com.br/), an open-access web platform designed to store Cq values, which allows users to visualize and compare reference gene stability data under different experimental conditions.

Validation of the reference gene was carried out by analyzing the expression profile of the target gene *IbAGPASE*, a gene involved in the regulation of starch synthesis and production of tuberous roots^31^. The *IbAGPASE* expression pattern was normalized with the three most and least stable reference genes, according to the classification generated by the RefFinder algorithm. The expression rate and corresponding confidence intervals were estimated using a linear mixed-effects model ^67^ implemented in the lme4 package ^68^. Model residuals were verified for normality and all plots were generated in R ^69^ with the ggplot2 package^70^.

## Supplementary Information

Below is the link to the electronic supplementary material.

Supplementary Material 1

Supplementary Material 2

## Data Availability

All data generated and analyzed for this study are included in this published article and its Supplementary Information file. All programs used to analyze the data are publicly available.
