# Supplementary material for: Evaluation and validation of reference genes for RT-qPCR normalization in different sweet potato tissues
Source: Sci Rep. 2025 Nov 14;15:39899. doi: 10.1038/s41598-025-22650-7 (PMC12618874; doi:10.1038/s41598-025-22650-7)
Supplement: Supplementary file 1 — Supplementary Material 1 [file 41598_2025_22650_MOESM1_ESM.pdf]

Evaluation and validation of reference genes for RT-qPCR normalization in different sweet potato tissues

Melissa Barbosa Fonseca Moraes[1,2]; Matheus Martins Daúde[1,2]; Kellen Kauanne Pimenta de Oliveira[1]; Rogério Cavalcante Gonçalves[1]; Solange Aparecida Ságio[1,3]; André Almeida Lima [1]; Antônio Chalfun-Junior[4]; Márcio Antônio da Silveira[3]; Horllys Gomes Barreto[1,2,3*]

[1]Laboratory of Molecular analysis (LAM), Life Sciences Department, Federal University of Tocantins, Palmas, TO, Brazil.

[2]Postgraduate Program in Biodiversity and Biotechnology, Rede Bionorte, Federal University of Tocantins, Palmas, TO, Brazil.

[3]Postgraduate Program in Digital Agroenergy, Federal University of Tocantins, Palmas, TO, Brazil.

[4]Plant Molecular Physiology Laboratory, Biology Department, Federal University of Lavras, Lavras, MG, Brazil.

***Corresponding author:**
Horllys Gomes Barreto
Laboratory of Molecular Analyses (LAM)
Department of Life Sciences
Federal University of Tocantins/UFT, University Campus of Palmas, TO, Brazil.
Postal Code: 77.001-090
E-mail: horllys@uft.edu.br

SUPPLEMENTARY S1

Table S1 Candidate reference gene expression levels, based on the Cq (Cycle of Quantification) data, obtained from roots (R), tuberous roots (T), stem (C), and leaves (F) from sweet potato (*Ipomoea batatas*) plants grown under natural conditions. Three biological replicates (numbers 1 to 3) were used, and reactions were run in triplicates as technical repetitions.

Treatment / Biological replicate	*IbCYC*	*IbPLD*	*IbACT*	*IbARF*	*IbRPL*	*IbGAP*	*IbTUB*	*IbUBI*	*IbCOX*	*IbEF1α*
R1	18.35	20.80	17.72	18.56	19.87	17.52	20.37	21.58	30.24	21.18
R1	18.35	20.87	17.73	18.45	19.78	17.46	20.48	21.53	30.56	21.52
R1	18.37	20.94	17.63	18.49	19.63	17.42	20.23	21.54	31.37	21.07
R2	19.02	22.48	18.97	19.65	22.75	18.55	22.48	24.12	30.82	22.17
R2	19.29	22.52	18.99	19.82	22.65	18.64	22.42	24.13	30.60	22.62
R2	19.21	22.61	18.99	19.52	22.68	18.62	22.32	24.15	30.74	22.14
R3	18.83	22.09	18.30	18.80	21.45	17.64	21.40	23.11	33.81	21.68
R3	18.77	22.01	18.24	18.87	21.49	17.67	21.45	23.17	33.56	21.47
R3	18.74	22.18	18.18	18.66	21.50	17.64	21.42	23.17	32.53	21.31
T1	18.92	21.73	18.84	19.41	21.49	19.24	21.31	21.78	28.73	20.62
T1	18.87	21.79	18.79	19.51	21.35	19.01	21.65	21.82	29.54	20.56
T1	18.88	21.89	18.80	19.34	21.52	19.26	21.74	21.96	29.38	20.26
T2	19.41	21.69	18.20	18.95	20.28	19.12	21.10	22.12	28.90	21.14
T2	19.39	21.70	18.18	18.92	20.27	19.02	21.22	22.12	29.98	21.19
T2	19.56	21.79	18.01	18.74	20.26	19.24	21.00	22.11	29.89	20.86
T3	18.11	20.80	18.57	19.12	21.90	18.69	21.05	21.24	29.38	20.61
T3	18.02	20.89	18.53	19.25	21.98	18.82	21.52	21.18	29.49	20.33

T3	18.21	20.85	18.51	19.18	21.91	18.67	21.65	21.26	29.74	20.77
C1	18.95	21.53	18.27	19.31	20.92	18.60	20.56	23.37	30.88	22.41
C1	18.96	21.61	18.19	19.24	20.82	18.47	20.43	23.45	29.71	22.73
C1	18.96	21.60	18.16	19.24	20.65	18.41	20.42	23.43	29.67	22.91
C2	18.34	20.90	17.83	18.73	20.72	18.02	20.06	22.65	28.89	22.30
C2	18.43	20.88	17.78	18.61	20.54	18.61	20.05	22.66	28.66	22.72
C2	18.42	20.90	17.70	18.56	20.55	18.31	20.05	20.64	28.97	22.57
C3	17.81	21.20	17.62	18.31	19.37	17.59	19.64	22.35	28.53	20.97
C3	17.92	21.30	17.51	18.14	19.38	17.45	19.40	22.28	28.75	21.97
C3	17.93	21.20	17.52	18.19	19.39	17.45	19.63	22.28	27.86	21.14
F1	20.11	23.23	19.46	20.67	18.25	20.94	21.37	23.52	29.16	22.96
F1	20.35	23.27	19.56	20.66	18.16	20.93	21.26	23.62	29.45	23.37
F1	20.15	23.14	19.43	20.58	18.14	20.90	21.16	23.61	28.79	23.27
F2	20.09	24.24	21.19	21.88	20.49	22.77	22.52	24.36	29.64	24.28
F2	20.35	24.56	21.03	21.91	20.52	22.78	22.42	24.46	29.26	24.40
F2	20.19	24.41	21.02	21.91	20.52	22.70	22.59	24.08	29.23	24.76
F3	21.81	25.45	21.31	22.53	19.64	23.50	22.85	26.02	29.21	24.22
F3	22.21	25.53	21.30	22.54	19.64	23.50	22.86	26.19	29.37	24.93
F3	21.94	25.62	21.22	22.60	19.72	23.54	22.84	26.10	29.45	24.99
**Mean**	**20,80**	**24,38**	**20,61**	**21,70**	**19,45**	**22,40**	**22,21**	**24,66**	**29,28**	**24,13**
**Standard deviation**	**0,90**	**1,01**	**0,85**	**0,85**	**1,02**	**1,15**	**0,73**	**1,13**	**0,24**	**0,76**

Table S2 Ranking of the candidate reference genes generated according to their stability values calculated by the geNorm, NormFinder, BestKeeper, Delta-Ct, and RefFinder algorithms using the Cq (Cycle of Quantification) data of the four sweet potato (*Ipomoea batatas*) tissues (fibrous roots, tuberous roots, stem, and leaves) combined, from plants grown under natural conditions.

Gene	geNorm		NormFinder		BestKeeper		Delta-Ct		RefFinder	
	Stability value (M)	Ranking	Stability value (SV)	Ranking	Stabilit y value (SD)	Ranking	Stabilit y value (ΔCt)	Rankin g	Stability value (SV)	Ranking
IbACT	0.234	1	0.261	1	0.930	4	0.915	1	1.414	1
IbARF	0.234	1	0.431	3	1.043	6	0.932	2	2.449	2
IbCYC	0.472	4	0.375	2	0.847	2	0.976	4	2.828	3
IbTUB	0.741	7	0.432	4	0.798	1	1.071	5	3.440	4
IbPLD	0.389	3	0.514	5	1.129	7	0.974	3	4.213	5
IbUBI	0.594	5	0.679	6	1.138	8	1.123	6	6.160	6
IbEF1α	0.674	6	0.842	7	1.144	9	1.201	7	7.172	7
IbCOX	1.239	10	1.735	9	0.930	3	1.902	10	7.208	8
IbRPL	1.073	9	1.736	10	0.934	5	1.899	9	7.977	9
IbGAP	0.818	8	1.238	8	1.521	10	1.398	8	8.459	10

Table S3 Ranking of the candidate reference genes generated according to their stability values calculated by the geNorm, NormFinder, BestKeeper, Delta-Ct, and RefFinder algorithms using the Cq (Cycle of Quantification) data of two sweet potato (*Ipomoea batatas*) tissues (fibrous roots and tuberous roots) from plants grown under natural conditions.

Gene	geNorm		NormFinder		BestKeeper		Delta-Ct		RefFinder	
	Stability value (M)	Rankin g	Stability value (SV)	Rankin g	Stability value (SD)	Rankin g	Stabilit y value (ΔCt)	Rankin g	Stability value (SV)	Ranking
IbACT	0.119	1	0.319	3	0.378	2	0.669	2	1.861	1
IbARF	0.119	1	0.346	4	0.353	1	0.678	3	1.861	2
IbTUB	0.257	3	0.239	2	0.474	4	0.666	1	2.213	3
IbPLD	0.384	4	0.181	1	0.525	6	0.683	4	3.130	4
IbCYC	0.447	5	0.459	5	0.382	3	0.773	5	4.401	5
IbEF1α	0.582	7	0.483	6	0.505	5	0.820	6	5.958	6
IbGAP	0.517	6	0.843	9	0.599	7	0.975	9	7.637	7
IbUBI	0.646	8	0.634	7	0.869	9	0.903	7	7.707	8
IbRPL	0.693	9	0.753	8	0.833	8	0.956	8	8.239	9
IbCOX	0.870	10	1.504	10	1.097	10	1.576	10	10.000	10

Table S4 Ranking of the candidate reference genes generated according to their stability values calculated by the geNorm, NormFinder, BestKeeper, Delta-Ct, and RefFinder algorithms using the Cq (Cycle of Quantification) data of two sweet potato (*Ipomoea batatas*) tissues (fibrous roots and stem) from plants grown under natural conditions.

Gene	geNorm		NormFinder		BestKeeper		Delta-Ct		RefFinder	
	Stability value (M)	Ranking	Stability value (SV)	Ranking	Stabilit y value (SD)	Rankin g	Stability value (ΔCt)	Rankin g	Stability value (SV)	Ranking
IbACT	0.184	1	0.092	1	0.403	2	0.567	1	1.189	1
IbCYC	0.195	3	0.186	2	0.378	1	0.599	3	2.060	2
IbARF	0.184	1	0.226	4	0.418	3	0.590	2	2.213	3
IbPLD	0.345	5	0.225	3	0.536	5	0.641	4	4.162	4
IbGAP	0.281	4	0.530	6	0.466	4	0.719	5	4.681	5
IbTUB	0.444	6	0.436	5	0.802	7	0.750	6	5.958	6
IbRPL	0.512	7	0.563	7	0.886	9	0.818	7	7.454	7
IbUBI	0.559	8	0.608	8	0.811	8	0.836	8	8.000	8
IbEFIα	0.601	9	0.752	9	0.574	6	0.880	9	8.132	9
IbCOX	0.799	10	1.539	10	1.311	10	1.594	10	10.000	10

Table S5 Ranking of the candidate reference genes generated according to their stability values calculated by the geNorm, NormFinder, BestKeeper, Delta-Ct, and RefFinder algorithms using the Cq (Cycle of Quantification) data of two sweet potato (*Ipomoea batatas*) tissues (fibrous roots and leaves) from plants grown under natural conditions.

Gene	geNorm		NormFinder		BestKeeper		Delta-Ct		RefFinder	
	Stability value (M)	Ranking	Stability value (SV)	Ranking	Stability value (SD)	Ranking	Stability value (ΔCt)	Ranking	Stability value (SV)	Ranking
IbACT	0.279	1	0.305	2	1.157	4	1.009	1	1.682	1
IbTUB	0.625	7	0.186	1	0.787	1	1.186	7	2.646	2
IbCYC	0.436	5	0.426	4	1.015	2	1.103	5	3.761	3
IbARF	0.279	1	0.617	7	1.359	9	1.063	4	3.984	4
IbPLD	0.346	4	0.501	5	1.275	8	1.050	2	4.229	5
IbUBI	0.523	6	0.383	3	1.042	3	1.133	6	4.243	6
IbEF1α	0.286	3	0.501	6	1.223	7	1.062	3	4.409	7
IbRPL	1.132	9	2.009	9	1.179	6	2.209	9	8.132	8
IbCOX	1.418	10	2.449	10	1.170	5	2.562	10	8.409	9
IbGAP	0.796	8	1.718	8	2.244	10	1.806	8	8.459	10

Table S6 Ranking of the candidate reference genes generated according to their stability values calculated by the geNorm, NormFinder, BestKeeper, Delta-Ct, and RefFinder algorithms using the Cq (Cycle of Quantification) data of two sweet potato (*Ipomoea batatas*) tissues (tuberous roots and stem) from plants grown under natural conditions.

Gene	geNorm		NormFinder		BestKeeper		Delta-Ct		RefFinder	
	Stability value (M)	Ranking	Stability value (SV)	Ranking	Stability value (SD)	Ranking	Stability value (ΔCt)	Ranking	Stability value (SV)	Ranking
IbARF	0.166	1	0.051	1	0.360	3	0.565	1	1.316	1
IbACT	0.166	1	0.260	2	0.356	2	0.593	2	1.682	2
IbPLD	0.400	4	0.356	3	0.356	1	0.670	4	2.632	3
IbGAP	0.306	3	0.365	5	0.465	4	0.638	3	3.663	4
IbCYC	0.426	5	0.361	4	0.484	5	0.671	5	4.729	5
IbCOX	0.513	7	0.468	6	0.546	6	0.742	6	6.236	6
IbTUB	0.464	6	0.603	7	0.667	9	0.752	7	7.172	7
IbRPL	0.563	8	0.730	8	0.664	8	0.865	8	8.000	8
IbUBI	0.662	9	0.840	9	0.586	7	0.986	9	8.452	9
IbEF1α	0.767	10	1.093	10	0.831	10	1.185	10	10.000	10

Table S7 Ranking of the candidate reference genes generated according to their stability values calculated by the geNorm, NormFinder, BestKeeper, Delta-Ct, and RefFinder algorithms using the Cq (Cycle of Quantification) data of two sweet potato (*Ipomoea batatas*) tissues (tuberous roots and leaves) from plants grown under natural conditions.

**Gene**	**geNorm**		**NormFinder**		**BestKeeper**		**Delta-Ct**		**RefFinder**	
	Stability value (M)	Rankin g	Stability value (SV)	Rankin g	Stability value (SD)	Rankin g	Stabilit y value (ΔCt)	Rankin g	Stability value (SV)	Ranking
IbARF	0.437	4	0.297	2	1.270	6	0.875	1	2.632	1
IbACT	0.541	6	0.135	1	1.085	5	0.900	2	2.783	2
IbPLD	0.254	1	0.638	5	1.462	7	0.936	3	3.201	3
IbUBI	0.254	1	0.721	6	1.465	8	0.990	4	3.722	4
IbTUB	0.727	8	0.497	3	0.597	2	1.096	7	4.281	5
IbCYC	0.606	7	0.510	4	0.991	4	1.019	5	4.865	6
IbCOX	0.913	9	1.260	9	0.254	1	1.526	9	5.196	7
IbGAP	0.362	3	0.890	7	1.694	9	1.074	6	5.803	8
IbRPL	1.178	10	2.183	10	0.962	3	2.237	10	7.401	9
IbEF1α	0.488	5	0.924	8	1.713	10	1.128	8	7.521	10

Table S8 Ranking of the candidate reference genes generated according to their stability values calculated by the geNorm, NormFinder, BestKeeper, Delta-Ct, and RefFinder algorithms using the Cq (Cycle of Quantification) data of two sweet potato (*Ipomoea batatas*) tissues (stem and leaves) from plants grown under natural conditions.

**Gene**	**geNorm**		**NormFinder**		**BestKeeper**		**Delta-Ct**		**RefFinder**	
	Stability value (M)	Rankin g	Stability value (SV)	Rankin g	Stability value (SD)	Rankin g	Stabilit y value (ΔCt)	Rankin g	Stability value (SV)	Ranking
IbTUB	0.391	4	0.171	1	1.091	5	0.770	1	2.115	1
IbACT	0.223	1	0.313	3	1.386	7	0.798	2	2.546	2
IbARF	0.223	1	0.411	5	1.497	8	0.807	3	3.310	3
IbEF1α	0.518	6	0.283	2	0.992	3	0.884	5	3.663	4
IbCYC	0.457	5	0.379	4	1.193	6	0.865	4	4.681	5
IbCOX	0.874	9	1.261	8	0.437	1	1.448	9	5.045	6
IbPLD	0.311	3	0.628	7	1.574	9	0.909	6	5.803	7
IbUBI	0.579	7	0.581	6	1.058	4	0.986	7	5.856	8
IbRPL	1.065	10	1.751	10	0.780	2	1.828	10	6.687	9
IbGAP	0.691	8	1.263	9	2.147	10	1.350	8	8.712	10

Table S9 Ranking of the candidate reference genes generated according to their stability values calculated by the geNorm, NormFinder, BestKeeper, Delta-Ct, and RefFinder algorithms using the Cq (Cycle of Quantification) data of three sweet potato (*Ipomoea batatas*) tissues (fibrous roots, tuberous roots and stem) from plants grown under natural conditions.

**Gene**	**geNorm**		**NormFinder**		**BestKeeper**		**Delta-Ct**		**RefFinder**	
	Stability value (M)	Rankin g	Stability value (SV)	Rankin g	Stability value (SD)	Rankin g	Stabilit y value (ΔCt)	Rankin g	Stability value (SV)	Ranking
IbACT	0.164	1	0.249	2	0.388	2	0.669	1	1.414	1
IbARF	0.164	1	0.252	3	0.387	1	0.673	2	1.565	2
IbPLD	0.406	4	0.247	1	0.487	4	0.713	3	2.632	3
IbCYC	0.361	3	0.351	4	0.426	3	0.736	4	3.464	4
IbGAP	0.466	5	0.687	6	0.560	5	0.886	6	5.477	5
IbTUB	0.517	6	0.504	5	0.727	7	0.805	5	5.692	6
IbRPL	0.584	7	0.693	7	0.853	9	0.927	7	7.454	7
IbUBI	0.676	8	0.743	8	0.803	8	0.984	8	8.000	8
IbEF1α	0.754	9	0.905	9	0.698	6	1.091	9	8.132	9
IbCOX	0.894	10	1.346	10	1.087	10	1.456	10	10.000	10

Table S10 Ranking of the candidate reference genes generated according to their stability values calculated by the geNorm, NormFinder, BestKeeper, Delta-Ct, and RefFinder algorithms using the Cq (Cycle of Quantification) data of three sweet potato (*Ipomoea batatas*) tissues (fibrous roots, tuberous roots and leaves) from plants grown under natural conditions.

**Gene**	**geNorm**		**NormFinder**		**BestKeeper**		**Delta-Ct**		**RefFinder**	
	Stability value (M)	Rankin g	Stability value (SV)	Rankin g	Stability value (SD)	Rankin g	Stabilit y value (ΔCt)	Rankin g	Stability value (SV)	Ranking
IbACT	0.252	1	0.261	1	0.984	3	0.985	1	1.316	1
IbARF	0.252	1	0.496	4	1.168	6	1.006	2	2.632	2
IbTUB	0.718	7	0.368	2	0.629	1	1.147	6	3.027	3
IbCYC	0.519	4	0.448	3	0.899	2	1.067	4	3.130	4
IbPLD	0.423	3	0.530	5	1.220	7	1.019	3	4.213	5
IbUBI	0.627	6	0.628	6	1.268	8	1.141	5	6.160	6
IbEF1α	0.580	5	0.741	7	1.338	9	1.150	7	6.853	7
IbRPL	1.128	9	1.976	9	1.092	5	2.138	9	7.770	8
IbCOX	1.334	10	1.992	10	0.984	4	2.157	10	7.953	9
IbGAP	0.818	8	1.383	8	1.750	10	1.531	8	8.459	10

Table S11 Ranking of the candidate reference genes generated according to their stability values calculated by the geNorm, NormFinder, BestKeeper, Delta-Ct, and RefFinder algorithms using the Cq (Cycle of Quantification) data of three sweet potato (*Ipomoea batatas*) tissues (tuberous roots, stem and leaves) from plants grown under natural conditions.

Gene	geNorm		NormFinder		BestKeeper		Delta-Ct		RefFinder	
	Stability value (M)	Ranking	Stability value (SV)	Ranking	Stability value (SD)	Ranking	Stability value (ΔCt)	Ranking	Stability value (SV)	Ranking
IbACT	0.238	1	0.230	1	1.087	5	0.873	2	1.778	1
IbARF	0.238	1	0.334	2	1.230	7	0.867	1	1.934	2
IbCYC	0.505	4	0.397	3	0.995	4	0.944	3	3.464	3
IbTUB	0.742	7	0.480	4	0.817	2	1.024	5	4.091	4
IbPLD	0.397	3	0.604	5	1.349	9	0.956	4	4.821	5
IbCOX	0.962	9	1.169	9	0.420	1	1.433	9	5.196	6
IbUBI	0.679	6	0.762	6	1.212	6	1.102	6	6.000	7
IbGAP	0.600	5	1.037	8	1.707	10	1.227	7	7.274	8
IbRPL	1.162	10	1.871	10	0.831	3	1.961	10	7.401	9
IbEF1α	0.814	8	0.940	7	1.313	8	1.231	8	7.737	10
