# Supplementary material for: Evaluation and validation of reference genes for RT-qPCR normalization in different sweet potato tissues
Source: Sci Rep. 2025 Nov 14;15:39899. doi: 10.1038/s41598-025-22650-7 (PMC12618874; doi:10.1038/s41598-025-22650-7)
Supplement: Supplementary file 2 — Supplementary Material 2 [file 41598_2025_22650_MOESM2_ESM.pdf]

## Evaluation and validation of reference genes for RT-qPCR normalization in different sweet potato tissues

Melissa Barbosa Fonseca Moraes[1,2]; Matheus Martins Daúde[1,2]; Kellen Kauanne Pimenta de Oliveira[1]; Rogério Cavalcante Gonçalves[1]; Solange Aparecida Ságio[1,3]; Antônio Chalfun-Junior[4]; Márcio Antônio da Silveira[3]; Horllys Gomes Barreto[1,2,3*]

[1]Laboratory of Molecular analysis (LAM), Life Sciences Department, Federal University of Tocantins, Palmas, TO, Brazil.

[2]Postgraduate Program in Biodiversity and Biotechnology, Rede Bionorte, Federal University of Tocantins, Palmas, TO, Brazil.

[3]Postgraduate Program in Digital Agroenergy, Federal University of Tocantins, Palmas, TO, Brazil.

[4]Plant Molecular Physiology Laboratory, Biology Department, Federal University of Lavras, Lavras, MG, Brazil.

### SUPPLEMENTARY S2

**Figure 1** *Melting* curves of the candidate reference genes analyzed.

***IbAGPase***

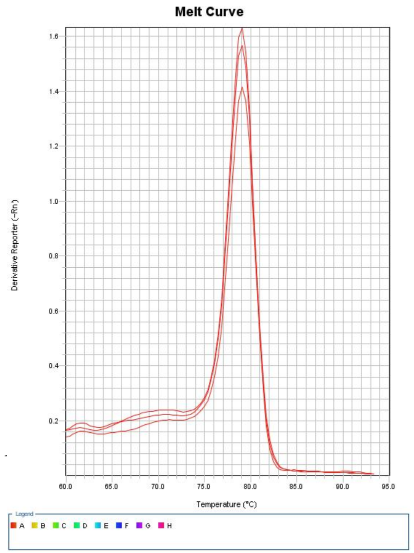


***IbACT***

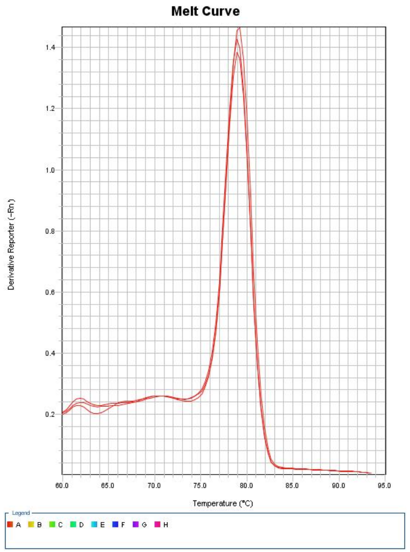


***IbARF***

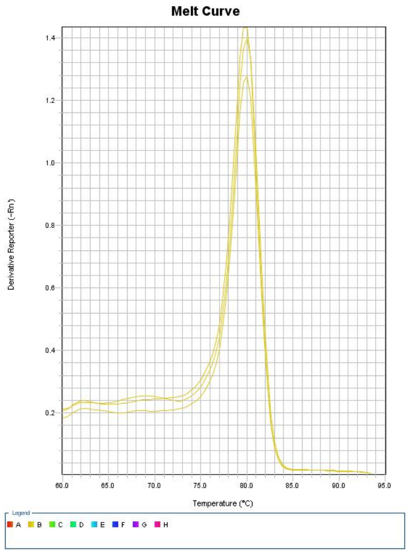

*IbCYC*

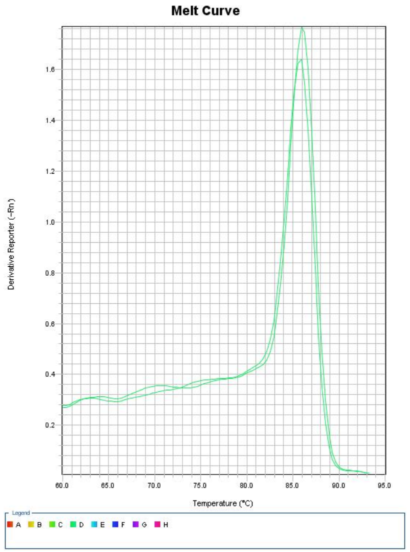


*IbPLD*

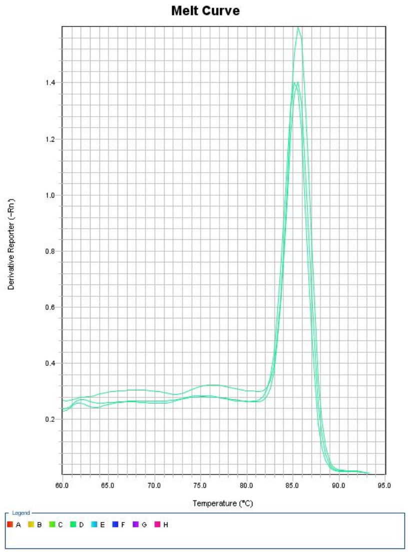


*IbEF1-α*

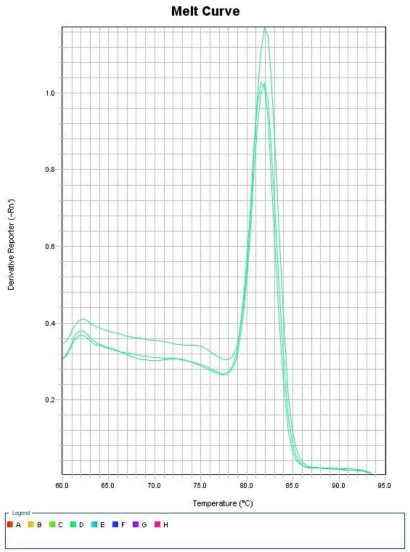


*IbTUB*

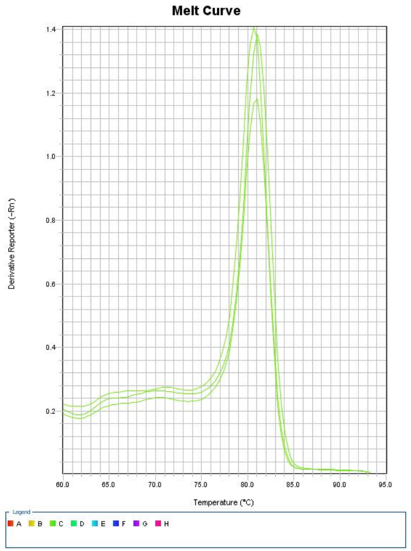


*IbUBI*

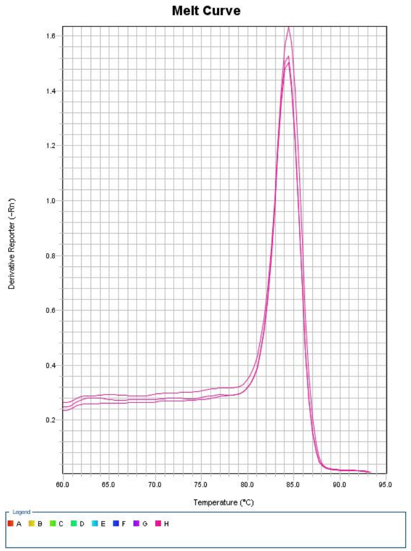


*IbRPL*

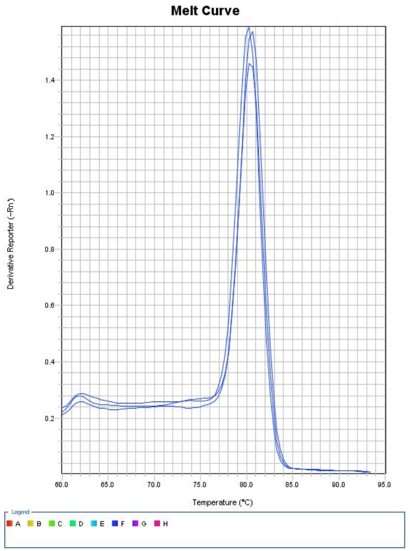

***IbCOX***

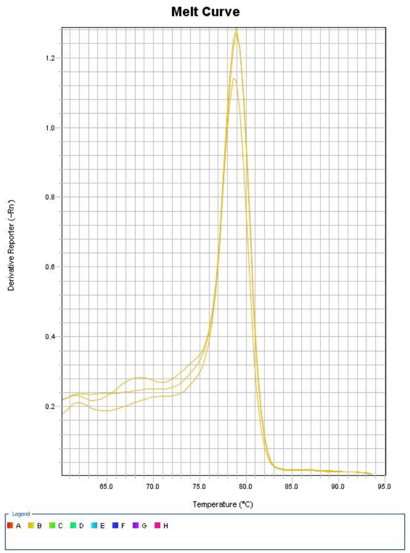
Melt Curve
Derivative Reporter (-Rn')
Temperature (°C)
Legend
A B C D E F G H

***IbGAP***

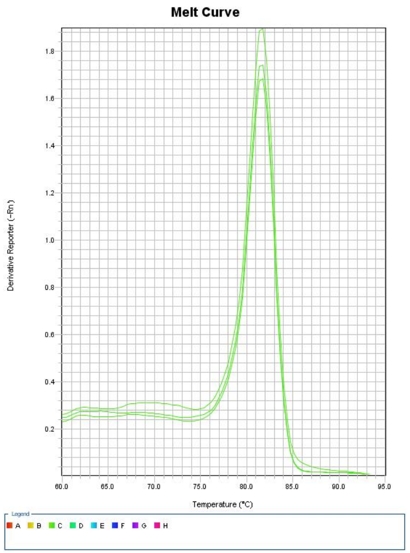
Melt Curve
Derivative Reporter (-Rn')
Temperature (°C)
Legend
A B C D E F G H
